# Inter-laboratory study on standardized MPS libraries: evaluation of performance, concordance, and sensitivity using mixtures and degraded DNA

**DOI:** 10.1007/s00414-019-02201-2

**Published:** 2019-11-19

**Authors:** Petra Müller, Christian Sell, Thorsten Hadrys, Johannes Hedman, Steffi Bredemeyer, Francois-Xavier Laurent, Lutz Roewer, Sabrina Achtruth, Maja Sidstedt, Titia Sijen, Marc Trimborn, Natalie Weiler, Sascha Willuweit, Ingo Bastisch, Walther Parson, the SeqForSTR-Consortium

**Affiliations:** 1 Institute of Legal Medicine, Medical University of Innsbruck, Müllerstraße 44, 6020 Innsbruck, Austria; 2 Federal Criminal Police Office, Wiesbaden, Germany; 3 Institute of Forensic Sciences, DNA Department, Bavarian State Criminal Police Office, Munich, Germany; 4 Swedish National Forensic Centre (NFC), Linköping, Sweden; 5 Applied Microbiology, Department of Chemistry, Lund University, Lund, Sweden; 6 Institute of Legal Medicine and Forensic Sciences, Charité – Universitätsmedizin Berlin, Berlin, Germany; 7 Institut National de Police Scientifique, Laboratoire de Police Scientifique de Lyon, Ecully Cedex, France; 8 The Police President in Berlin, Forensic Science Institute, Berlin, Germany; 9 Biological Traces, Netherlands Forensic Institute, Laan van Ypenburg 6, 2497 GB The Hague, The Netherlands; 10 Forensic Science Program, The Pennsylvania State University, State College, PA USA

**Keywords:** Short tandem repeats, Massively parallel sequencing, ForenSeq DNA Signature Prep Kit, Inter-laboratory study, Collaborative study

## Abstract

We present results from an inter-laboratory massively parallel sequencing (MPS) study in the framework of the SeqForSTRs project to evaluate forensically relevant parameters, such as performance, concordance, and sensitivity, using a standardized sequencing library including reference material, mixtures, and ancient DNA samples. The standardized library was prepared using the ForenSeq DNA Signature Prep Kit (primer mix A). The library was shared between eight European laboratories located in Austria, France, Germany, The Netherlands, and Sweden to perform MPS on their particular MiSeq FGx sequencers. Despite variation in performance between sequencing runs, all laboratories obtained quality metrics that fell within the manufacturer’s recommended ranges. Furthermore, differences in locus coverage did not inevitably adversely affect heterozygous balance. Inter-laboratory concordance showed 100% concordant genotypes for the included autosomal and Y-STRs, and still, X-STR concordance exceeded 83%. The exclusive reasons for X-STR discordances were drop-outs at DXS10103. Sensitivity experiments demonstrated that correct allele calling varied between sequencing instruments in particular for lower DNA amounts (≤ 125 pg). The analysis of compromised DNA samples showed the drop-out of one sample (FA10013B01A) while for the remaining three degraded DNA samples MPS was able to successfully type ≥ 87% of all aSTRs, ≥ 78% of all Y-STRs, ≥ 68% of all X-STRs, and ≥ 92% of all iSNPs demonstrating that MPS is a promising tool for human identity testing, which in return, has to undergo rigorous in-house validation before it can be implemented into forensic routine casework.

**Electronic supplementary material:**

The online version of this article (10.1007/s00414-019-02201-2) contains supplementary material, which is available to authorized users.

## Introduction

With the emergence of massively parallel sequencing (MPS) technologies, molecular genetic tools have been developed to characterise the nucleotide sequence of short tandem repeat (STR) markers that have so far been analysed using fragment sizing by capillary electrophoresis (CE) [1–7]. As is common practise in forensic genetics, such novel tools undergo detailed evaluation [3, 8–12] and validation [1, 13, 14] prior to their application in casework. Recent population studies have shown that sequencing of STRs lead to an increased power of discrimination compared to commonly used CE sizing by a) illustrating micro-variant structures and b) identifying sequence variations located in the repeat- (isometric variants; alleles of identical size but different in internal sequence) or flanking region [15–20]. Moreover, current MPS-kits provide the option to multiplex various loci simultaneously within one reaction, such as STRs (autosomal, Y- and X-chromosomal), mitochondrial DNA (control region) as well as single nucleotide polymorphisms (SNPs) that provide estimates of identity, phenotypic traits, biogeographical and ancestry information [21–24]. The inclusion of SNPs adds the benefit [25–27] that these markers allow for shorter amplicon design, which can support human identification of degraded or challenging DNA samples [28–30]. Although published studies have demonstrated that MPS is a promising tool for forensic applications, little is known about the method’s variability and potential differences in the limit of detection between sequencing platforms from the same supplier. Inter-laboratory studies are however highly relevant to understand the performance of this new technology when applied to real casework settings. Since the workflow for MPS-based sequence analysis of forensic genetic markers is more complex than for electrophoresis-based systems, we decided to reduce the complexity of this study to libraries prepared at the organising laboratory (OL) from forensically relevant samples that were shipped to the participating laboratories in the following way: In the framework of the SeqForSTRs project (EU Project ISF-Nr. IZ25-5793-2015-30 2017-2020, [31]) the ForenSeq DNA Signature Prep Kit [22] was used for collaborative validation experiments and population studies. As a general benchmark for the comparability of the results between laboratories, and, to reduce the inherent complexity of MPS experiments derived from multiple steps during manual library preparation, the SeqForSTRs consortium performed a collaborative exercise analysing a centrally prepared, standardized sequencing library. The library was distributed between eight participating laboratories located in Austria, France, Germany, The Netherlands, and Sweden and were analysed on their respective MiSeq FGx sequencing platforms (Illumina, San Diego, USA; [32]). In order to monitor the effect of shipment, one library aliquot was sent to one of the participants, immediately returned to the OL and analysed (run 8) to compare the results to those of the test run prior to shipment (run 0).

We note that only STR genotype calls were considered here in agreement with the aim of the SeqForSTRs project, except for ancient DNA samples, where also SNP information was analysed to evaluate their performance in highly degraded DNA. To rate the performance of iSNPs in compromised samples additional SNP information was examined for standard reference material 2391c (components A–C). The library contained selected DNA samples (control DNAs, reference material, mixtures and degraded DNA) to test for instrument variability, concordance, and sensitivity.

## Materials and methods

This collaborative study combined data generated in nine sequencing runs that were conducted in eight laboratories located in Austria, France, Germany, The Netherlands, and Sweden (Table 1). To ensure uniform starting conditions a standardized sequencing library consisting of 25 selected DNA samples, a negative and a positive amplification control (see “Sample selection” section) was prepared using the ForenSeq DNA Signature Prep Kit (Verogen, San Diego, USA) at the OL. To assess the quality of sequencing products an initial test run (run 0) was performed at the OL (Table 1) prior to shipment. Pooled sequencing libraries were shipped with the United Parcel Service (unchilled and under ambient conditions) and sequenced by all participants using their respective MiSeq FGx sequencers (Illumina). To monitor transport conditions (under ambient temperatures) one aliquot of the library was shipped by the OL to one of the participants, returned to the OL upon receipt and analysed (run 8).

**Table 1:** Run and quality metrics information for all sequencing runs performed within the SeqforSTRs study. The resulting data were analysed and presented in anonymous form. An initial test run (run 0) was performed at the organising laboratory to assess the quality of sequencing products. Prior to sequencing each laboratory loaded 7 μL of pooled library on the MiSeq FGx instrument according to the manufacturer’s recommendation (Verogen)

Run	Cluster density (K/mm2)	Cluster passing filter (%)	Phasing (%)	Pre-phasing (%)	Total no. of reads (performance testing)	UAS version	NTC
UAS Guide (range)	400–1650 K/mm2	≥ 80%	≤ 0.25%	≤ 0.15%	Single source samples		
0	706	96.13	0.141	0.102	704,959	1.2.16337	Negative
1	684	97.71	0.180	0.099	847,323	1.2.16337	Negative
2	432	97.25	0.168	0.144	438,786	1.2.16337	Negative
3	818	94.79	0.171	0.103	948,395	1.2.16173	Negative
4	603	97.65	0.193	0.084	641,752	1.2.16337	Negative
5	587	96.19	0.166	0.113	579,248	1.2.16337	Negative
6	486	97.76	0.226	0.102	650,416	1.2.16173	Negative
7	862	93.86	0.181	0.100	1,123,576	1.2.16337	Negative
8	625	97.03	0.192	0.130	729,121	1.2.16337	Negative
Mean	645	96.49	0.185	0.109	740,397		
SD	141	1.38	0.020	0.018	205,448		
Median	614	97.03	0.181	0.102	689,769		
Min	432	93.86	0.141	0.084	438,786		
Max	862	97.76	0.226	0.144	1,123,576		

### Sample selection

#### Concordance, mixture, and sensitivity

Concordance was assessed using three single donor standard reference materials (SRM): 2391c, component A–C [33] purchased form the National Institute of Standards and Technology (NIST; Gaithersburg, USA), control DNA 2800M (Promega, Madison, USA), and two samples from previous GEDNAP (German DNA Profiling; https://www.gednap.org/) proficiency tests (G49-S4, single source; G49-S1, two-person mixture).

Two-person mixtures were prepared using control DNAs 2800M (major contributor; Promega) and 9947A (minor contributor; Thermo Fisher Scientific (TFS), Waltham, USA) at a stock concentration of 9 ng/μL and five ratios (M500 = 1:1; M166 = 83.3:16.7; M91 = 90.9:9.1; M62.5 = 93.7:6.3 and M47.6 = 95.2:4.8). The initial intention was to prepare the mixtures with a male minor contributor however, the female fraction (9947A) was used as minor contributor mistakenly, which affected the ability to test male minor contributions in the mixture analyses. All mixtures were diluted accordingly to 1 ng/μL in molecular grade water prior to library preparation. Mixture study consisted of M166, M91, M62.5 and M47.6, which were analysed as singletons only. Sensitivity was assessed using mixture M500 prepared to a final DNA input of 1000 pg, 500 pg, 250 pg, 125 pg, 62 pg and 31 pg in molecular grade water and analysed in duplicate.

#### Ancient DNA samples

To determine the robustness and performance of large MPS panels on highly degraded DNA, four ancient DNA (aDNA) samples were included. Libraries were prepared from extracted DNA from two bone (femur: FA10013B01A, humerus: FA10026B01A) and two tooth samples (molar: FA10030T01A, incisor: FA10058T01B) according to [22], respectively. Ancient samples dated between the fifth to eighth century ([34]) and were taken from earlier studies [35].

### DNA quantification

The amount of genomic DNA was determined with a real-time PCR assay targeting specific AluYb8 sequences [36]. A spiked in vitro mutagenized and cloned part of the *human retinoblastoma susceptibility protein 1* (*RB1*) gene was co-amplified as internal amplification positive control (pRB1_IPC_) according to [37], updated in [35]. Calibration curve analysis covered a DNA input range from 10 ng to 169.5 fg per reaction and was analysed in duplicate. The reaction volume was 10 μL consisting of 5 μL TaqMan Fast Universal PCR mix (TFS), 3 μL primer probe premix (made in-house) and 2 μL extracted DNA sample. Thermal cycler protocol consisted of an initial denaturation at 95 °C for 20 s followed by 40 cycles of denaturation at 95 °C for 3 s and annealing/elongation at 60 °C for 30 s. Samples were run on an Applied Biosystems 7500 Fast Real-Time PCR Instrument (TFS) using the HID Real-Time PCR Software v 2.3. Kinetic information for the pRB1_IPC_ system yielded no indication of inhibition during DNA amplification.

### Library preparation and sequencing

Each laboratory obtained one 2 mL screw-cap tube (Sarstedt, Nümbrecht, Germany; labelled: *SeqForSTRs, SeqPool 20 μL, ForenSeq Kit, 29.11.2017 PM*) containing 20 μL pooled sequencing library. The final library pool was made of 25 selected DNAs, a negative and a positive amplification control. To ensure sufficient library volume OL simultaneously prepared three library pools, including the same samples and index adapters (i5 and i7) using the ForenSeq DNA Signature Prep Kit, primer mix A (Verogen, [22]) in one 96-well plate (Table S1). Library preparation, purification and normalization were performed according to [22]. All samples were amplified with 1 ng DNA according to [22], except for G49-S4 (2.6 ng DNA), the samples for the sensitivity study (serial dilution: 1000 pg–31 pg DNA) and aDNA samples.

Prior to pooling, 30 μL of each library sample (present in pools 1, 2 and 3; Table S1) was joined into a 0.8 mL deep-well storage plate (one sample/well). Libraries were pooled, diluted and denatured following [22] before loading into a MiSeq FGx Reagent Cartridge and sequenced on the MiSeq FGx instrument ([32], Table S2). To achieve uniform designation for all sequencing runs OL provided a text-based sample sheet including relevant information on, e.g. sample name as well as adapter combination needed for demultiplexing and data analysis (Table S3).

### Data analysis

#### Capillary electrophoresis

STR typing was performed at OL with PowerPlex ESX 17 (Promega [38]), PowerPlex 16 System (Promega [39]), PowerPlex 21 System (Promega [40]), Investigator Argus X-12 (Qiagen, Hilden, Germany [41]), AmpFlSTR Yfiler (TFS [42]), Genderplex (an in-house developed sex-typing assay [35, 43]) or AmpFlSTR NGM SElect Kit (TFS [44]). Amplification was performed on an Applied Biosystems GeneAmp 9700 thermal cycler (TFS) following the manufacturer’s or recommended protocols [35, 38–44]. PCR fragments were separated and detected using an Applied Biosystems Prism 3500XL Genetic Analyzer (TFS). The analysis of size-based STR fragments was conducted at OL with GeneMapper ID-X software, version 1.2 (TFS) by applying in-house validated dye thresholds: blue – 50 relative fluorescence units (RFU), green – 80 RFU, yellow – 100 RFU, red – 100 RFU.

#### Universal Analysis Software

Analysis of MPS data utilized the ForenSeq Universal Analysis Software, version 1.2 (Verogen, exact version see Table 1) for allele and genotype calling by applying the manufacturer’s default analysis settings [45]. Universal Analysis Software (UAS) called alleles and genotypes based on the number of reads by applying a specified analytical (AT; 1.5%) and interpretation threshold (IT; 4.5%), except for DYS398II (AT: 5%; IT: 15%), DYS448 (AT: 3.3%; IT: 10%) and DYS635 (AT: 3.3%; IT: 10%) [45]. Single nucleotide polymorphism (SNP) analysis of aDNA samples were manually revised and no-call genotype corrected according to [11]. For example, no-call SNPs that fell below the manufacturer’s default IT but in the range of 20 to 29 reads were manually corrected and genotype results put in brackets. No-call SNPs that fell below the manufacturer’s default IT but showed reads ≥ 30 were manually corrected and called. All laboratories applied UAS using the preinstalled default settings. Following to sequencing, an Excel-based genotype report-file was generated at each laboratory and sent to the OL for further analysis. Provided data were analysed at OL and reported in anonymous form.

### Statistical analyses

Figures, diagrams and statistical analyses were generated using Microsoft Excel, GraphPad Prism, version 7.04 for Windows and IBM SPSS, version 24 [46].

### Allele frequencies and statistical calculations

Allele frequencies used for genotype frequency calculations were taken from SPSmart v5.1.1 [47–50]. Allele frequencies for SRM 2391c (components A–C) [33] were selected according to the manufacturer’s provided information on geographic origin of the respective DNA sample (2391cA: European, 2391cB: American, 2391cC: Oceania). Random match probability (RMP) estimates were calculated according to the formulae 4.1. [51]. Alternatively, likelihood ratios (LR) were used, which is a measure of the power of proof regarding the hypothesis that two DNA profiles were derived from the same suspect and is known as the inverse of the RMP [51, 52].

## Results and discussion

### Sequencing run information

The results of the collaborative exercise were analysed using UAS, which provided the following general information on the sequencing runs (recommended values in brackets [45]; Table 1): cluster density (400–1650 K/mm^2^), cluster passing filter (≥ 80%), phasing (≤ 0.25%) and pre-phasing (≤ 0.15%). Mean cluster density plus standard deviation (SD) was calculated for all runs and amounted on average to 645 K/mm^2^ (SD = 141 K/mm^2^) with a minimum of 432 K/mm^2^ (run 2) and a maximum of 862 K/mm^2^ (run 7; Table 1). The library was analysed at OL after preparation (run 0) and after shipment (run 8). The cluster density of the post-shipment run (run 8) yielded 88.5% of the initial cluster density value (run 0). Still, this cluster density fell within the range of the cluster densities of the other sequencing runs. Mean values (SD in brackets) for cluster passing filter, phasing and pre-phasing amounted on average to 96.49% (1.38%), 0.185% (0.020%) and 0.109% (0.018%) with a minimum of 93.86% (run 7), 0.141% (run 0) and 0.084% (run 4) and a maximum of 97.76% (run 6), 0.226% (run 6) and 0.144% (run 2), respectively (Table 1). Based on these findings, we conducted a more detailed analysis of all datasets to examine possible effects of cluster density variation on the overall data interpretation quality. Below, we present and discuss inter-laboratory concordance (Inter-laboratory concordance section), locus coverage (Locus coverage section) and heterozygous balance (Heterozygous balance section). All negative amplification controls yielded no detectable sequences.

### Inter-laboratory concordance

Inter-laboratory concordance was assessed by comparing a) genotype profiles to known reference profiles (SRM 2391c A–C, control DNA 2800M) [22, 33] or previously obtained MPS results (GEDNAP samples G49-S1 and G49-S4; data not shown) and b) genotype from identical library preparation and run in different laboratories. All runs obtained fully concordant autosomal STR (aSTR) and Y-STR results to known reference and between sequencing runs for SRM 2391c A–C, control DNA 2800M, G49-S1 and G49-S4 (Table S4). X-STR analysis revealed full concordance to known reference and between sequencing runs for all runs, except for runs 1 (G49-S1), 2 (2391cA, 2391cC), 4 (2800M) and 6 (2800M, G49-S1). X-STR concordance for control DNA 2800M, SRM 2391cA, 2391cC and G49-S1 was 85.7% (runs 4 and 6: 6 out of 7 alleles; both runs), 92.3% (run 2: 12 out of 13 alleles), 85.7% (run 2: 6 out of 7 alleles) and 91.7% (runs 1 and 6: 11 out of 12 alleles; both runs), respectively (Table S4). All six instances of discordances were due to allelic drop-out at DXS10103 (control DNA 2800M: allele 18 (hemizygous genotype); SRM 2391cA: allele 19 (genotype: 18, 19); SRM 2391cC: allele 19 (hemizygous genotype) and G49-S1: allele 18 (genotype: 18, 20) (Table S4)). Interestingly, for G49-S1 runs 0, 2–5 and 8 obtained a marker drop-out at DX10103 resulting in 83.3% concordance, while only run 7 correctly typed both alleles that were known from reference (Table S4). In addition, DXS10103 was reported to be sensitive to locus drop-out in earlier studies [1, 8, 13, 53, 54] most likely due to underperformance of this marker during amplification. Therefore, further optimization would be required as recommended by [16, 53].

### Performance testing

Cluster density is known to be an important but also critical parameter that affects overall run quality, the total amount of sequencing reads and reads passing filter. For example, while lower cluster density does not necessarily adversely affect data quality, it predominantly leads to decreased data output. In contrast, overclustering potentially leads to poor run performance including the introduction of sequencing artefacts, elevated sequencing error rates together with lowered sequencing data output [55]. Based on the observed cluster density variation (Table 1, “Sequencing run information” section) we checked for possible differences in locus coverage and intra-locus balance (heterozygous genotype). The sample set analysed in this respect consisted of five single source samples (control DNA 2800M, SRM 2391c A–C and GEDNAP G49-S4).

#### Locus coverage

Locus coverage was calculated for each STR marker and sequencing run (Table S5). Among aSTRs, TH01 (49,387; 13,501) showed the highest mean number of reads while D19S443 (3691; 1003) performed the least in terms of locus coverage (Table S5). In line with these findings for the ForenSeq DNA Signature Prep Kit, Müller et al., 2018 [4] observed comparable results for TH01 and D19S443 evaluating both Globalfiler MPS-kits (i.e. the Early Access Applied Biosystems Precision ID Globalfiler Mixture ID and the Globalfiler NGS STR Panels) on the Ion S5 instrument [4]. Although using a centrally prepared sequencing library we observed locus coverage variation for single source samples sequenced on the eight MiSeq FGx sequencers (Tables 1 and S5). The mean number of reads/run was 740,397 (205,448) ranging from 438,786 reads (run 2) to 1,123,576 reads (run 7) relating to the afore-mentioned single source samples (Table 1). Run 2 yielded lowest locus coverage over all STRs, whereas run 7 displayed highest locus coverage for nearly all STRs (55 out of 59 STRs; 93.2%) (Table S5).

#### Heterozygous balance

Heterozygous balance (HB), also known as intra-locus balance, was calculated (for each sample and locus) for heterozygous- and isometric genotypes by dividing the lower number of allele reads by the higher number of allele reads [56]. Average HB/run and mean HB/locus was calculated for 27 aSTRs, Amelogenin, two Y-STRs and six X-STRs. Results were comparable for each particular locus between all sequencing runs (Table S6). All markers showed HB ≥ 0.60 (manufacturer’s default threshold settings) except for D22S1045 and D5S818 that yielded lower HB values, 0.43 (0.02) and 0.57 (0.02), respectively (Table S6). These findings were in line with earlier studies that reported imbalances at D22S1045 [3, 12–15, 17, 22, 54, 57] and D5S818 [53, 54, 57]. Six aSTRs (D4S2408, CSF1PO, D7S820, D8S1179, D9S1122 and TH01), one Y-STR (DYF387S1) and three X-STRs (DXS10135, DXS10074 and HPRTB) displayed HB ≥ 0.90 (Table S6). These findings were consistent with previous studies [3, 13]. Results for afore-mentioned aSTRs and one X-STR were in line with Silvia et al., 2017 [54], except for DXS10135 and DXS10074.

**Fig. 1:**
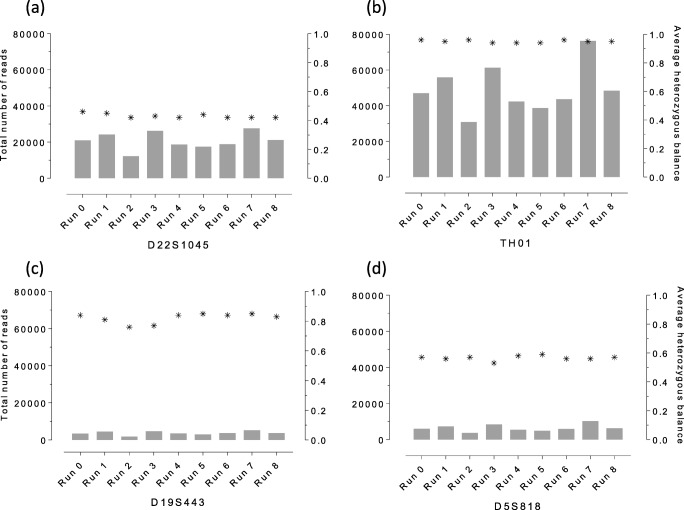
Inter-laboratory investigation of possible correlations between locus coverage differences and heterozygous imbalances. Total locus coverage and average heterozygous balance (HB) were calculated using five single source samples (SRM 2391c A-C, control DNA 2800M and G49-S1; amplified with 1 ng DNA). Against expectations, we found that varying locus coverage did not affect heterozygous balance. For example, **a** D22S1045 and **d** D5S818 were found to show HB ≤ 0.60 but mean locus coverages of 20,838 reads (D22S1045) and 6460 reads (D5S818). In contrast, **b** TH01 and **c** D19S443 were found to be highly balanced forensic markers though showing obvious differences in locus coverage (mean locus coverage of 49,387 reads (TH01) and 3691 reads (D19S443); Table S5)

Apparently, this analysis showed that low locus coverage did not necessarily increase heterozygous imbalance (Fig. 1). Among aSTRs, TH01 showed highest locus coverage along with highly balanced allele calls (0.95, 0.01). In contrast, D19S443 displayed lowest locus coverages for most sequencing runs with relatively balanced genotype calls (0.82, 0.03) (Table S6, Fig. 1). Observed differences in locus coverage as a result of cluster density variation had only minor effects on the fluctuation of HB (Tables 1 and S6).

### Sensitivity

Sensitivity of the ForenSeq DNA Signature Prep Kit was assessed with decreasing DNA input of sample M500 ranging from 1000 pg to 31 pg (final DNA input). Alleles were called based on read counts when they exceeded the manufacturer’s default IT [1, 45]. The full profile of M500 consisted of 80 aSTR alleles, 26 Y-STR alleles and 17 X-STR alleles.

The mean aSTR sensitivity success rate over all runs was 100% down to 500 pg and still ≥ 91.8% (2.6%) down to 62 pg DNA input (Table S7). On average, all sequencing runs successfully typed ≥ 93.2% (2.8%) of all Y-STRs down to 125 pg DNA (Table S7). Furthermore, X-STR analysis showed sensitivity levels ≥ 90.5% (2.5%) using 125 pg DNA (Table S7). For all runs, the average sensitivity level was 71.0% (5.1%), 60.3% (5.8%) and 81.1% (4.2%) for aSTRs, Y-STRs and X-STRs, respectively, using 31 pg DNA (Table S7). These findings were in agreement with Churchill et al., 2016 [3] who reported yields of more than 94% of all alleles using 100 pg DNA amplified with the ForenSeq DNA Signature Prep Kit.

**Fig. 2:**
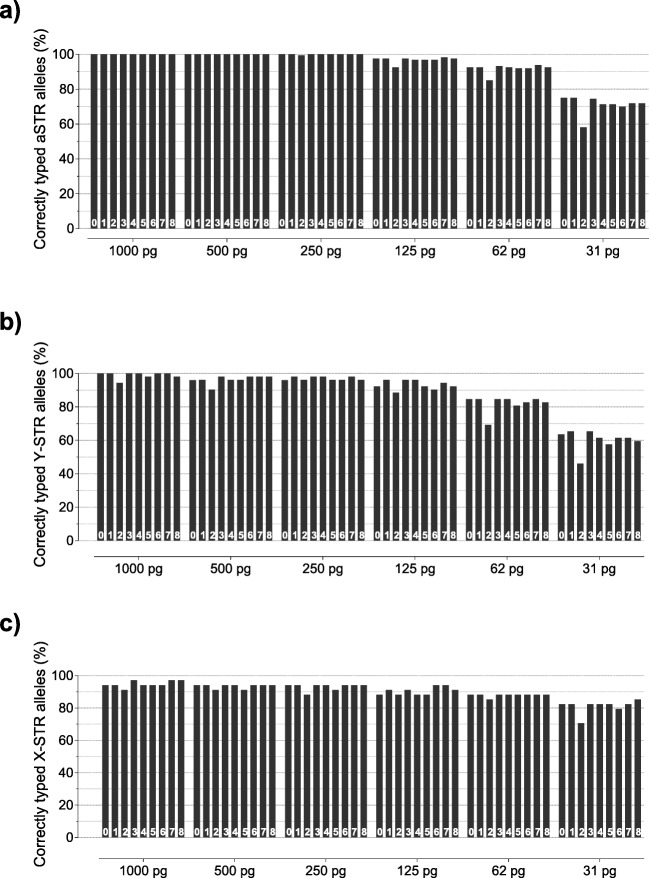
Sensitivity summary showing sensitivity levels for each sequencing run (numbers on the bottom indicate the respective sequencing run) and marker class included in the ForenSeq DNA Signature Prep Kit: **a** autosomal STRs (aSTRs), **b** Y-STRs, and **c** X-STRs. Sample M500 was prepared at ratio 1:1 using control DNAs 9947A and 2800M. A serial dilution was prepared at 1000 pg, 500 pg, 250 pg, 125 pg, 62 pg, and 31 pg total DNA input and amplified in duplicate. Results indicate that all sequencing runs obtained comparable sensitivity levels for all classes of markers, except for run 2. Particularly for DNA input amounts of ≤ 62 pg sensitivity levels of run 2 differed distinctly from the other sequencing runs

To investigate potential instrument-related differences in sensitivity we carefully examined aSTR, Y-STRs and X-STR results for each sequencing run individually. Results for aSTRs revealed that all runs obtained full-profiles down to 250 pg, except for run 2 (drop-out at D19S443, allele 13, for 250 pg) (Table S8, Fig. 2a). The results showed that all runs obtained comparable sensitivity levels for all classes of markers included in the ForenSeq DNA Signature Prep Kit, except for run 2 (Table S8, Figs. 2 and S1). Particularly for lower DNA input amounts (≤ 62 pg) sensitivity levels of run 2 differed distinctly from the others (Fig. 2a–c). Still, run 2 success rates were 58.1%, 46.2% and 70.6% for aSTRs, Y-STRs and X-STRs using as little as 31 pg DNA, respectively (Table S7, Figs. 2 and S1). In general, results for the ForenSeq DNA Signature Prep Kit aSTR sensitivity were comparable to CE-based STR kits [58–61] and in line with earlier published MPS studies [1, 3, 4, 14, 54].

### Mixtures

We defined mixtures here as DNA profiles showing more than two alleles in at least two forensic STR markers [62], based on biological material from two or more persons contributing to the sample being tested [63]. Mixture study was performed using samples M166, M91, M62.5 and M47.6 prepared from control DNAs 9947A (minor contributor) and 2800M (major contributor) at four ratios (83.3:16.7; 90.9:9.1; 93.7:6.3 and 95.2:4.8). The total DNA input amount for each mixture was 1 ng. Within each mixture we identified aSTR alleles that were consistent with the minor contributor genotype (9947A). Note, only those 12 aSTRs with genotype calls that were distinguishable from the major contributor’s genotype were included for mixture analysis (D1S1656, TPOX, D2S1338, D3S1358, D5S818, D8S1179, vWA, PentaE, D16S539, D18S51, D21S11 and D22S1045) yielding a total of 41 distinguishable alleles (Table 2 a and b). Alleles were called based on their reads by applying the manufacturer’s default thresholds. On average, the ForenSeq DNA Signature Prep Kit was able to detect 100%, 94.4% (1.7%), 76.7% (5.0%) and 45% (5.0%) of the minor contributor’s alleles for M166, M91, M62.5 and M47.6, respectively (Table 2b). Furthermore, for M62.5 and M47.6 we observed a noticeable decrease in the proportion of correctly assigned alleles (Figure S2a), which is most likely related to the decline in minor contributor’s DNA input, while the major contributors DNA input remained almost unchanged (Table 2a, Figure S2b). Referring to M47.6 (ratio: 95.2:4.8) we were able to type 73.2% (2.4%) of all alleles. These findings for mixture analysis were comparable to Jäger et al., 2017 [1], who observed on average 73 allele calls out of 83 alleles (or 87.9%) at 5% minor contributor DNA input including 27 aSTRs. However, when detecting the minor contributors alleles, findings for M91 demonstrated that 5% variation (ranging from 90% to 95%) was obtained between all sequencing runs, which increased to 15% for M62.5 (ranging from 65% to 80%) and M47.6 (ranging from 40% to 55%), respectively (Table 2b, Figure S2a). Still, results for M47.6 showed that on average all runs were able to detect 45% (5%) of the minor contributor’s alleles using as little as 47.6 pg DNA (Table 2b).

**Table 2:**
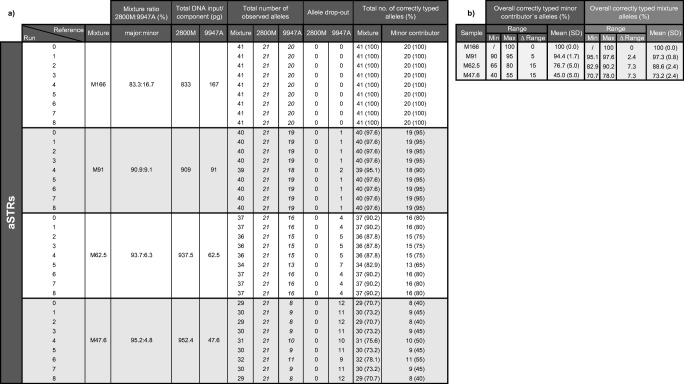
Summary of mixture analysis showing the total number of alleles observed for mixtures M166, M91, M62.5, and M47.6 prepared at four ratios (83.3:16.7; 90.9:9.1; 93.7:6.3, and 95.2:4.8). Mixtures were prepared using control DNAs 9947A (minor component) and 2800M (major component) amplified with 1 ng total DNA (analysed as singletons). Including only distinguishable autosomal STRs, we were able to identify 41 potential alleles per mixture at forensic markers D1S1656, TPOX, D2S1338, D3S1358, D5S818, D8S1179, vWA, PentaE, D16S539, D18S51, D21S11, and D22S1045. **a** depicts results obtained for all sequencing runs individually while **b** summarizes the average percentage of correctly typed alleles per minor contributor (dark grey header) and mixture (light grey header)

Again, despite the constant DNA input of 1 ng, clear differences in the total number of sequencing reads were obtained between the participating laboratories (Figure S2b). Figure S2b shows the read-proportion for each mixture component of M166, M91, M62.5, and M47.6 in relation to DNA input. As expected, reads of the minor component decreased with lowered DNA input while those of the major component accumulated with increasing DNA amount, except for M47.6. Generally, in proportion to DNA input the fraction of the minor contributor 9947A received higher number of reads compared to those of 2800M (Table S9). The observed mixture ratios were equivalent for all mixtures and between sequencing runs, except for M47.6, run 4 (Table S9). Although all mixtures were prepared using 1 ng DNA M47.6 showed an additional drop in the total number of reads for control DNA 2800M. However, this drop approximated the theoretical mixture ratio that would have been expected (Table S9, Figure S2b). It should be noted that the decrease in sequencing reads of control DNA 2800M is more likely related to sample preparation or laboratory procedure than to differences between sequencing instruments.

### Ancient DNA samples

The analysis of aDNA is associated with possible contamination at various stages of the process, some of which are difficult to control, e.g. contamination prior to receiving the sample in the laboratory. Also, aDNA is characterised by some degree of degradation that depends on various factors such as the general age of the specimen - although this is not following a strictly linear correlation - and environmental (storage) conditions. Usually ancient samples contain lower DNA quantity compared to contemporary samples [64]. To gather information on the ForenSeq DNA Signature Prep Kit’s performance on highly degraded DNA samples we included four compromised specimens from the early medieval times. As expected, we observed lowered percentage of correctly typed loci compared to high quality samples with broad variation in success rates between the samples (success rates ranged from 1.3% to 96.7%, Table 3, Figure S3). Genotypes were called when they exceeded the manufacturer’s default threshold [45]. Manual revision of the non-called STR loci (automatically by the software) was performed only for aDNA samples (all runs) if the locus information in the provided sample-details-report Excel-table indicated “INC” (genotype) in combination with the comment “interpretation threshold” (QC indicator). INC (inconclusive) indicated ambiguous genotype results that were not reported in the Excel-table. Non-called SNPs that indicated “INC” (genotype) in combination with “interpretation threshold” (QC indicator) and fell in the range of 20 to 29 reads were manually corrected and genotype results put in brackets (Universal Analysis Software section). Furthermore, the manual revision of homozygous STR genotype calls that indicated the comment “interpretation threshold” (QC indicator) was performed as described above, except for alleles in stutter position.

**Table 3:** Summary of ancient DNA analysis using the ForenSeq DNA Signature Prep Kit amplified for 27 autosomal STRs (aSTRs), 24 Y-STRs, 7 X-STRs, and 94 identity SNPs (iSNPs). The sample set consisted of four compromised samples dating from the fifth to eighth century (for more details see Table S10). Concordance was assessed by comparing genotype results between sequencing runs and to reference profile (if available, see Tables S10-S12). Overall markers indicate the mean percentage (standard deviation [SD] in brackets) of concordantly typed markers for each ancient DNA sample. The average number of typed markers per sample was calculated based on the mean percentage of concordantly typed markers. The maximum number of markers using the ForenSeq DNA Signature Prep Kit (primer mix A) is 152

Sample	Successfully typed markers per MPS run (%)										Successfully typed markers over all runs		
	Marker	Run									Mean (SD) [%]	Mean no. of typed markers/sample	No. of typed markers vs. total no. of markers
		0	1	2	3	4	5	6	7	8			
FA10013B01A	*aSTRs*	0	0	0	0	0	0	0	0	0	0	/	2/152
	*Y-STRs*	0	0	0	0	0	0	0	0	0	0	/	
	*X-STRs*	0	0	0	0	0	0	0	0	0	0	/	
	*iSNPs*	2.1	2.1	1.1	2.1	2.1	1.1	1.1	2.1	1.1	1.7 (0.5)	2	
FA10026B01A	*aSTRs*	92.6	96.3	85.2	96.3	92.6	88.9	92.6	92.6	92.6	92.2 (3.4)	25	136/151^a^
	*Y-STRs*	79.2	83.3	66.7	83.3	83.3	79.2	75.0	79.2	79.2	78.7 (5.3)	19	
	*X-STRs*	71.4	71.4	57.1	71.4	71.4	71.4	57.1	71.4	71.4	68.2 (6.3)	5	
	*iSNPs*	93.6	94.6	92.5	93.6	92.5	93.6	92.5	93.6	94.6	93.5 (0.8)	87	
FA10030T01A	*aSTRs*	85.2	88.9	81.5	92.6	88.9	88.9	81.5	88.9	88.9	87.3 (3.8)	24	116/128^b^
	*Y-STRs*	♀	♀	♀	♀	♀	♀	♀	♀	♀	♀	/	
	*X-STRs*	85.7	85.7	71.4	85.7	85.7	71.4	85.7	85.7	85.7	82.5 (6.3)	6	
	*iSNPs*	92.6	92.6	86.2	93.6	92.6	92.6	91.5	93.6	92.6	92.0 (2.3)	86	
FA10058T01B	*aSTRs*	96.3	100	96.3	100	100	96.3	96.3	96.3	100	97.9 (2.0)	26	147/152
	*Y-STRs*	100	100	95.8	100	100	100	95.8	100	100	99.1 (1.9)	24	
	*X-STRs*	85.7	85.7	85.7	85.7	85.7	85.7	85.7	85.7	85.7	85.7 (0.0)	6	
	*iSNPs*	96.8	97.8	95.7	97.9	96.8	96.8	98.9	97.9	96.8	97.3 (0.9)	91	

^**a**^Exclusion of rs722290 from concordance calculation due to tri-allelic genotype; therefore, the total number of markers is 151 instead of 152

^**b**^Sample derived from a female individual; therefore, the total number of markers recovered with the ForenSeq DNA Signature Prep Kit was 128 instead of 152

On average, the results for the ancient samples FA10013B01A, FA10026B01A, FA10030T01A, and FA10058T01B demonstrated that MPS was able to recover 2 out of 152 markers (1.3%), 136 out of 151 markers (90.1%), 116 out of 128 markers (90.6%) and 147 out of 152 markers (96.7%), respectively (Table 3, Figure S3).

The lowest percentage of correctly typed loci was obtained for FA10013B01A dating from the seventh–eighth century showing drop-out rates of 98.7% (150 out of 152 markers failed amplification) (Tables 5 and S10–S12, Figure S3). Only identity SNPs (iSNP) rs1109037 (genotype: GG; all runs) and rs6444724 (genotype: CC; runs 0, 1, 3, 4, and 7) were successfully typed. Manual revision of non-called iSNP genotype calls was performed as described in “Universal Analysis Software” section for rs6444724 (genotype: CC; runs 5, 6, and 8), rs740598 (genotype: A; for runs 1, 6; genotype: G; for run 7) and rs964681 for run 1 (genotype: TT) (Table S13).

The DNA profile of FA10026B01A was concordant between all runs and to known allele calls derived from CE (if available), except for D3S1358 (CE: 15; MPS: 14, 15), D12S391 (CE: 19; MPS: 19, 19.3), D21S11 (CE: 30; MPS: 29, 30) and DYS385a-b (CE: allele 14; MPS: 11, 14) (Tables S10–S11). The careful revision of previously generated CE data for sample FA10026B01A indicated peaks at D3S1358, D12S391, D21S11 and DYS385 that were either located in stutter position and/or fell below the in-house analytical threshold of 50 RFU. Based on these findings, and, because of the comparison of MPS and CE data, the most likely reason for the observed differences is allele drop-out. Results for all runs at locus D13S317 revealed the presence of three different isometric variants of allele 11, viz. (a) sequence identical to the reference sequence as taken from the updated “Forensic STR Sequence Guide v4” file [65] originally published in [66] and available online at https://strider.online [67], (b) allele 11 containing rs9546005-T, and (c) allele 11 containing a C>A transversion at nucleotide position GRCh38 CHR13:82148040 together with rs9546005-T, respectively (Tables S10 and S14). Furthermore, run 3 showed two isomeric variants of DYS385a-b, allele 11 (a) sequence identical to the reference sequence as taken from [65, 67], and (b) allele 11 containing a G>A transition located at GRCh38 CHRY:18639770 (18639770-G showing 107 reads and 18639770-A showing 38 reads), respectively (Table S11). A possible reason for sequence variants in degraded samples is based on the misincorporation of nucleotides during enzymatic amplification due to cytosine deamination during DNA degradation [68–71]. One instance of discordance between sequencing runs was found for run 2 showing allelic drop-out of the longer allele at D19S443 (MPS: 13, 14) (Table S10). Identity SNP results for FA10026B01A were fully concordant between sequencing runs, except for rs1015250 and rs722290 (Table S13). Manual revision of rs1015250 no-call iSNP revealed a potential G allele for runs 3–7 showing reads that accounted for 20%, 16%, 16%, 18%, and 14% of the called iSNP-allele reads, respectively. However, non-called iSNP, allele G, was found for rs1015250 for runs 0–2 and 8 too with reads ≤ 19. Data analysis revealed one tri-allelic genotype (iSNP genotype: C, G, (A)) at rs722290 for runs 0, 1 and 7. Therefore, rs722290 was excluded from concordance evaluation (Table S13). Observed reads for rs722290, allele A, added up to 4.6%, 4.2%, and 5.6% of allele-reads with highest intensity for runs 0, 1, and 7, respectively (data not shown). Locus drop-out was found for all runs at rs13182883, rs354439, rs719366, and at rs13218440 (runs 0, 2, 3, 5, and 6), rs1736442 (runs 0, 2, 4 and 5), and rs7041158 (run 2) (Table S13). The average mean over all runs for FA10026B01A was 92.2% (3.4%), 78.7% (5.3%), 68.2% (6.3%), and 93.5% (0.8%) of typed aSTRs, Y-STRs, X-STRs and iSNPs, respectively (Table 3, Figure S3).

Results for FA10030T01A were fully concordant between runs and to known reference (if available), except for D7S820 (CE: 8; MPS: 8, 10), D19S433 (CE: 12; MPS: 12, 13). Results for D22S1045 revealed the drop-out of the longer allele within all runs, except for run 3 (CE: 16, 17) (Table S10). In addition, all runs showed drop-out of the shorter allele at DXS8378 (CE: 11, 13) (Table S12). According to previously obtained CE results and to [34], human remains of FA10030T01A were derived from a female individual. Data analysis revealed one Y-STR call at DYS391, allele 11, most likely due to contamination during handling of the sample or laboratory procedure (Table S11). However, the total number of allele-reads for FA10030T01A at DYS391, allele 11, was ≤ 49 (Table S11). Identity SNP results for FA10030T01A were fully concordant between runs, except for rs1294331, rs354439, and rs1382387 showing the drop-out of allele A, allele T and allele G, respectively (Table S13). Locus drop-out was observed at rs2920816 (all runs), rs719366 (runs 0, 2–8), rs13182883 (runs 0–2, 4, 6–8) and rs13218440 (runs 1–6, 8). Overall, profile of FA10030T01A consisted of 87.3% (3.8%), 82.5% (6.3%), and 92.0% (2.3%) concordantly typed aSTRs, X-STRs, and iSNPs, respectively (Table 3, Figure S3).

The highest percentage of correctly typed loci was found for FA10058T01B, which was the eldest aDNA sample, dating from the fifth century showing 97.9% (2.0%), 99.1% (1.9%), 85.7% (0.0%), and 97.3% (0.9%) of concordantly typed aSTRs, Y-STRs, X-STRs, and iSNPs, respectively. Results were fully concordant between runs and to known reference (if available) for all 152 markers (Tables S10–S13, Figure S3). Drop-out was obtained for runs 2 and 5 at rs13218440 (Table S13). In addition, MPS was able to identify homozygous genotype as isometric heterozygous genotype for FA10058T01B at D8S1179, allele 13 (repeat structure variants [TCTA] [TCTG] [TCTA]_11_ and [TCTA]_13_ containing a G>A transition located at GRCh38 CHR8:124894872) and DYF387S1, allele 38 (repeat structure variants [AAAG]_3_ [GTAG] [GAAG]_4_ [AAAG]_2_ [GAAG] [AAAG]_2_ [GAAG]_9_ [AAAG]_8_ [AAAA] [AAAG]_7_ and [AAAG]_3_ [GTAG] [GAAG]_4_ [AAAG]_2_ [GAAG] [AAAG]_2_ [GAAG]_10_ [AAAG]_7_ [AAAA] [AAAG]_7_ both containing a G>A transition located at GRCh38 CHRY:23785484) (Table S14) [65, 67]. Earlier studies reported sequence variants at D8S1179 [4, 15, 72, 73], which was in line with our findings for this marker. However, at the time of writing, no references were found for DYF387S1. Discordances between sequencing runs were observed for three iSNPs at rs1355366, rs13182883, and rs13218440 due to the drop-out of allele G, allele A and allele A, respectively (Table S13). During manual revision of non-called iSNPs we observed reads that were below 1.8% of the called iSNP-allele reads at rs251934 (allele T) and rs1821380 (allele A) and were thus considered as noise. Runs 2 and 5 obtained locus drop-out at rs13218440 (Table S13).

In general, our results support the findings of earlier publications [74–76] that the application of MPS for STR analysis of degraded and challenging DNA samples can be beneficial. As expected and due to the nature of SNP-containing amplicons [25, 26], the ancient DNA data showed that, in contrast to STRs, the majority of sequencing runs obtained constantly high SNP recovery rates, which was in line with previous reports [8, 75] (Table 3). To evaluate the validity of the obtained results and to assess the impact of iSNPs vs. aSTRs we calculated the RMPs and LRs for each aDNA sample (if applicable) as well as for reference material SRM 2391c, components A–C (Tables S15–S18). RMPs for iSNPs were calculated from a subset of 49 iSNP that are included in the validated SNPforID 52-plex panel [77]. Note that rs1355366 was excluded for RMP calculation due to the ambiguous genotype results. The calculated RMPs for 27 aSTRs and 49 iSNPs for aDNAs ranged from 1.65E-32 to 7.15E-27 and from 1.37E-29 to 8.55E-28, respectively (Tables S15–S17), whereas the corresponding RMP values for SRM 2391c A–C ranged from 3.54E-43 to 2.48E-34 (aSTRs) and from 9.89E-31 to 1.40E-28 (iSNPs) (Table S18). As expected, the data of the reference material SRM 2391c A–C showed clearly higher LR levels for aSTRs than for the iSNP subset (Figure S4) [52, 78–80]. In the aDNA samples, however, the STR-based LRs were roughly in the same range as those obtained for iSNPs. Furthermore, there was no pronounced difference in iSNPs LR levels between reference and aDNA samples (Figure S4) [25, 52]. This relative decline of the STR-based LRs compared to the constant levels of SNP-based LRs can be an indication for a noticeably better performance of SNPs compared to STRs when analysing old and compromised DNA samples with MPS approaches. However, it is important to mention that the LR for SRM 2391cB was markedly higher than those of components A and C representing an above average LR value and that only a small number of compromised samples were included. Therefore, these data can be regarded as preliminary results indicating that SNPs are a preferable choice of markers, especially for heavily degraded DNA. Nevertheless, this has to be shown in more comprehensive MPS-studies.

Additionally, we contrasted CE-based with MPS-based aDNA genotyping. For this comparison, we calculated LRs (if applicable) for the AmpFlSTR NGM SElect Kit, the AmpFlSTR NGM SElect loci as included in the ForenSeq DNA Signature Prep Kit and for the entire set of aSTR as well as for a subset of 49 iSNP loci included in the latter kit (Tables S15–S17). The results clearly show the benefit of the large number of markers included in the MPS-based assay that resulted in much higher LRs than CE did (Tables S15–S17). In general, we observed comparable MPS-based LRs for all aDNA samples and classes of markers suggesting stable performance of the ForenSeq DNA Signature Prep Kit on compromised samples (Figure S5). Overall, the predominant reason for STR discordance between MPS and CE results within aDNA samples was due to additional allele calls obtained using MPS that lead to an increased power of discrimination, except for FA10013B01A, which showed total drop-out in MPS analysis but a partial profile using CE (Tables S10–S12). Possible reasons could be that the CE analysis was performed eight years earlier and the remaining DNA extract meanwhile frozen, stochastic effects due to low DNA input in general (Table S10) or inhibition of the MPS approach as described in [28, 81]. For instance, using MPS the average profile of FA10026B01A and FA10058T01B was composed of 25 aSTRs, 19 Y-STRs, 5-XSTRs and 87 iSNPs and 26 aSTRs, 24 Y-STRs, 6 X-STRs and 91 iSNPs, respectively (Table 3, Figure S5). However, CE analysis was able to recover 4 aSTRs, 5 Y-STRs and 15 aSTRs, 12 Y-STRs plus 1 X-STR for FA10026B01A and FA10058T01B, respectively (Tables S10–S11, Figure S5). Therefore, it seems important to note that such high numbers of different loci cannot be typed with CE-based STR kits within a single run. This indicates the usefulness of MPS-based technologies for the analysis of compromised samples especially if the original material is limited.

## Conclusions

The presented results are the first MPS-data collected in a collaborative exercise performed among eight independent European laboratories using sequencing instruments of the same supplier. This inter-laboratory study conducted in the framework of the SeqForSTRs project [31] described validation experiments for MPS STR analysis using a standardized sequencing library prepared with the ForenSeq DNA Signature Prep Kit and sequenced on eight different MiSeq FGx instruments. The primary intention of this study was to reduce the inherent complexity derived from multiple steps during manual library preparation to a minimum to test for instrument variation about which knowledge is scarce (including the effect of transport conditions). This was enabled by centralized library preparation using the ForenSeq DNA Signature Prep Kit (primer mix A). Despite broad observed variation in instrument performance, all laboratories obtained run quality metrics that fell within the manufacturer’s recommended range. Importantly, obtained locus coverage differences did not necessarily adversely affect heterozygous balances. Inter-laboratory concordance revealed 100% concordance for autosomal- and Y-STRs and yet 85.7% for X-STRs due to drop-out of one allele plus 83.3% due to locus drop-out at marker DXS10103. Mean success rates of all sensitivity runs with 125 pg DNA input were 96.9% (1.7%), 93.2% (2.8%), and 90.5% (2.5%) for aSTR, Y-STR, and X-STR alleles and were comparable to [1, 3, 53, 54] as well as to results using MPS-kits from other suppliers [9, 82]. Interestingly, sensitivity results for each particular run showed that especially for DNA input amounts of < 125 pg the ability to correctly type a STR profile might be dependent on the particular detection limit of the sequencing instrument. Sensitivity results showed that between laboratories the variation in successfully typed aSTRs (highest vs. lowest number of correctly typed alleles) increased with decreasing DNA input and added up to 9% (62 pg) and 17% (31 pg), respectively. These results indicate the importance of performing rigorous in-house validation before implementing MPS into forensic casework applications. Results showed that MPS is a promising tool for the identification of compromised DNA samples and were in line with [8, 75]. The analysis of degraded DNA samples using the ForenSeq DNA Signature Prep Kit showed the total drop-out of FA10013B01A; however, MPS was able to successfully type ≥ 87% of all aSTRs, ≥ 78% of all Y-STRs, ≥ 68% of all X-STRs, and ≥ 92% of all iSNPs for the remaining three compromised DNA samples. Again, this high number of successfully typed loci was not achieved using conventional CE-based technologies.

## Electronic supplementary material

Fig. S1 (PDF 302 kb)

Fig. S2 (PDF 435 kb)

Fig. S3 (PDF 124 kb)

Fig. S4 (PDF 78 kb)

Fig. S5 (PDF 82 kb)

Table S1 (XLSX 314 kb)
