# Supplementary material for: Inter-laboratory study on standardized MPS libraries: evaluation of performance, concordance, and sensitivity using mixtures and degraded DNA
Source: Int J Legal Med. 2019 Nov 19;134(1):185–98. doi: 10.1007/s00414-019-02201-2 (PMC6949318; doi:10.1007/s00414-019-02201-2)
Supplement: Supplementary file 1 — (PDF 302 kb) [file 414_2019_2201_MOESM1_ESM.pdf]

a)

	1000 pg	500 pg	250 pg	125 pg	62 pg	31 pg
Run 0	160	160	160	156	148	120
Run 1	160	160	160	156	148	120
Run 2	160	160	159	148	136	93
Run 3	160	160	160	156	149	119
Run 4	160	160	160	155	148	114
Run 5	160	160	160	155	147	114
Run 6	160	160	160	155	147	112
Run 7	160	160	160	157	150	115
Run 8	160	160	160	156	148	115

b)

	1000 pg	500 pg	250 pg	125 pg	62 pg	31 pg
Run 0	52	50	50	48	44	33
Run 1	52	50	51	50	44	34
Run 2	49	47	50	46	36	24
Run 3	52	51	51	50	44	34
Run 4	52	50	51	50	44	32
Run 5	51	50	50	48	42	30
Run 6	52	51	50	47	43	32
Run 7	52	51	51	49	44	32
Run 8	51	51	50	48	43	31

c)

	1000 pg	500 pg	250 pg	125 pg	62 pg	31 pg
Run 0	32	32	32	30	30	28
Run 1	32	32	32	31	30	28
Run 2	31	31	30	30	29	24
Run 3	33	32	32	31	30	28
Run 4	32	32	32	30	30	28
Run 5	32	31	31	30	30	28
Run 6	32	32	32	32	30	27
Run 7	33	32	32	32	30	28
Run 8	33	32	32	31	30	29

100 %
96-99 %
91-95 %
81-90 %
71-80 %
61-70 %
51-60 %
0-50 %

Figure S1. Sensitivity study heat maps for **a)** 27 aSTRs, **b)** 24 Y-STRs and **c)** 7 X-STRs included in the ForenSeq DNA Signature Prep Kit (Verogen). Numbers in cells depict the total number of alleles observed (two replicates) for each sequencing run that were correctly assigned to M500 depending on the DNA input. M500 was prepared at ratio 1:1 with control DNAs 2800M and 9947A. Serial dilution was prepared from 1000 pg down to 31 pg DNA input. Color gradient indicates the percentage of successfully called alleles from 100% (intensive green; full-profile) down to ≤ 50% (light-grey). Maximum number of alleles per replicate was 80, 26 and 17 for autosomal STRs (**a**), Y-STRs (**b**) and X-STRs (**c**), respectively.

Müller P., Sell C., Hadrys T., Hedman J., Bredemeyer S., Laurent F.X., Roewer L., Achtruth S., Sidstedt S., Sijen T., Trimborn M., Weiler N., Willuweit S., Bastisch I., Parson W., and the SeqForSTR-Consortium. Inter-laboratory study on standardized MPS libraries: Evaluation of performance, concordance and sensitivity using mixtures and degraded DNA
