# Supplementary material for: Inter-laboratory study on standardized MPS libraries: evaluation of performance, concordance, and sensitivity using mixtures and degraded DNA
Source: Int J Legal Med. 2019 Nov 19;134(1):185–98. doi: 10.1007/s00414-019-02201-2 (PMC6949318; doi:10.1007/s00414-019-02201-2)
Supplement: Supplementary file 2 — (PDF 435 kb) [file 414_2019_2201_MOESM2_ESM.pdf]

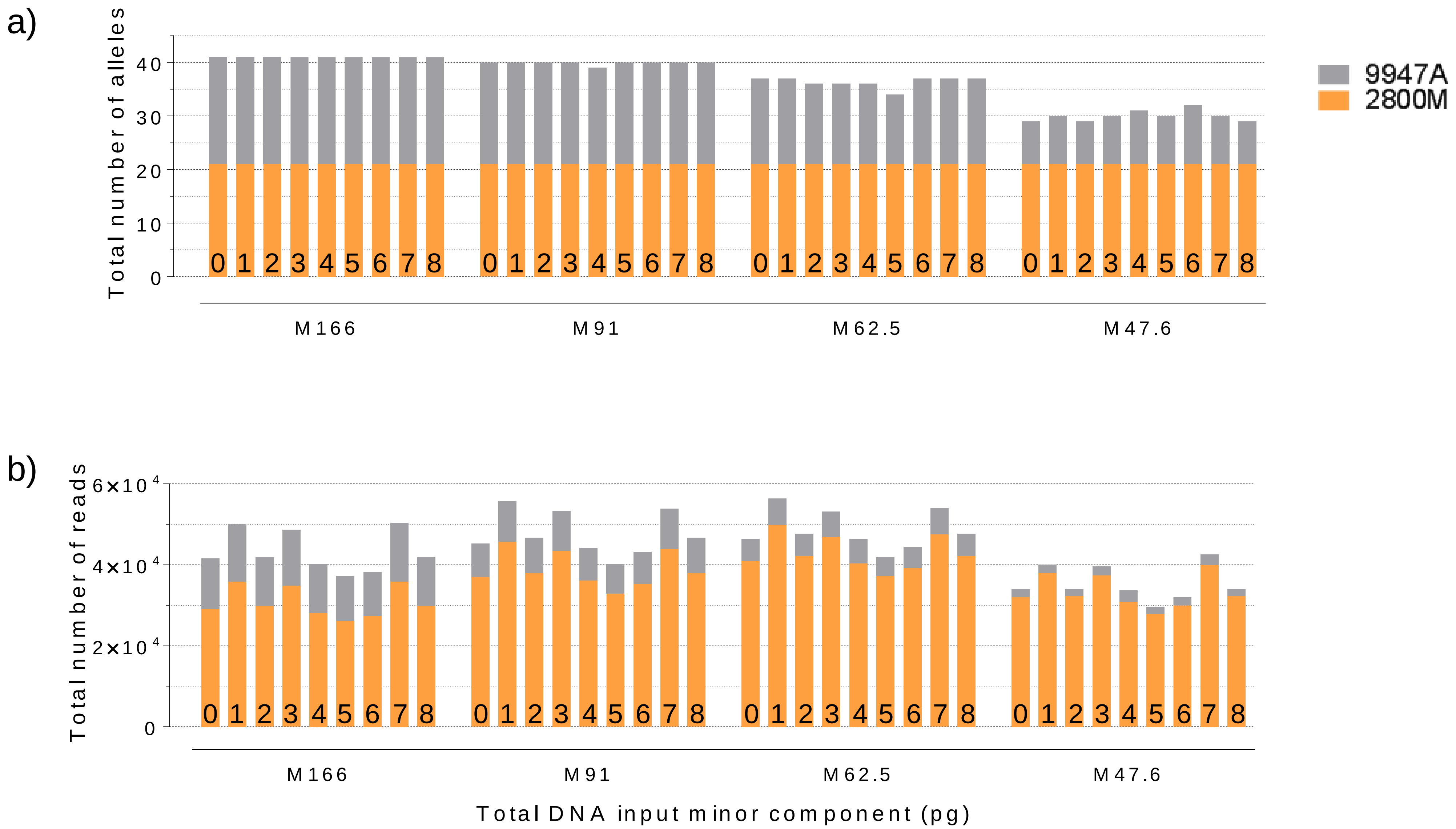


**Figure S2.** Total number of a) correctly typed alleles and b) observed reads/mixture-component. Mixtures M166, M91, M62.5 and M47.6 were prepared at ratios 83.3:16.7, 90.9:9.1, 93.7:6.3 and 95.2:4.8 using control DNAs 9947A (minor contributor) and 2800M (major contributor) and amplified with 1 ng total DNA input. Only STR markers that were distinguishable from one another were included for analysis (D1S1656, TPOX, D2S1338, D3S1358, D5S818, D8S1179, vWA, PentaE, D16S539, D18S51, D21S11 and D22S1045). Numbers on the bottom of each bar denote the associated sequencing run.

Müller P., Sell C., Hadrys T., Hedman J., Bredemeyer S., Laurent F.X., Roewer L., Achtruth S., Sidstedt S., Sijen T., Trimborn M., Weiler N., Willuweit S., Bastisch I., Parson W., and the SeqForSTR-Consortium. Inter-laboratory study on standardized MPS libraries: Evaluation of performance, concordance and sensitivity using mixtures and degraded DNA
