# Supplementary material for: Inter-laboratory study on standardized MPS libraries: evaluation of performance, concordance, and sensitivity using mixtures and degraded DNA
Source: Int J Legal Med. 2019 Nov 19;134(1):185–98. doi: 10.1007/s00414-019-02201-2 (PMC6949318; doi:10.1007/s00414-019-02201-2)
Supplement: Supplementary file 3 — (PDF 124 kb) [file 414_2019_2201_MOESM3_ESM.pdf]

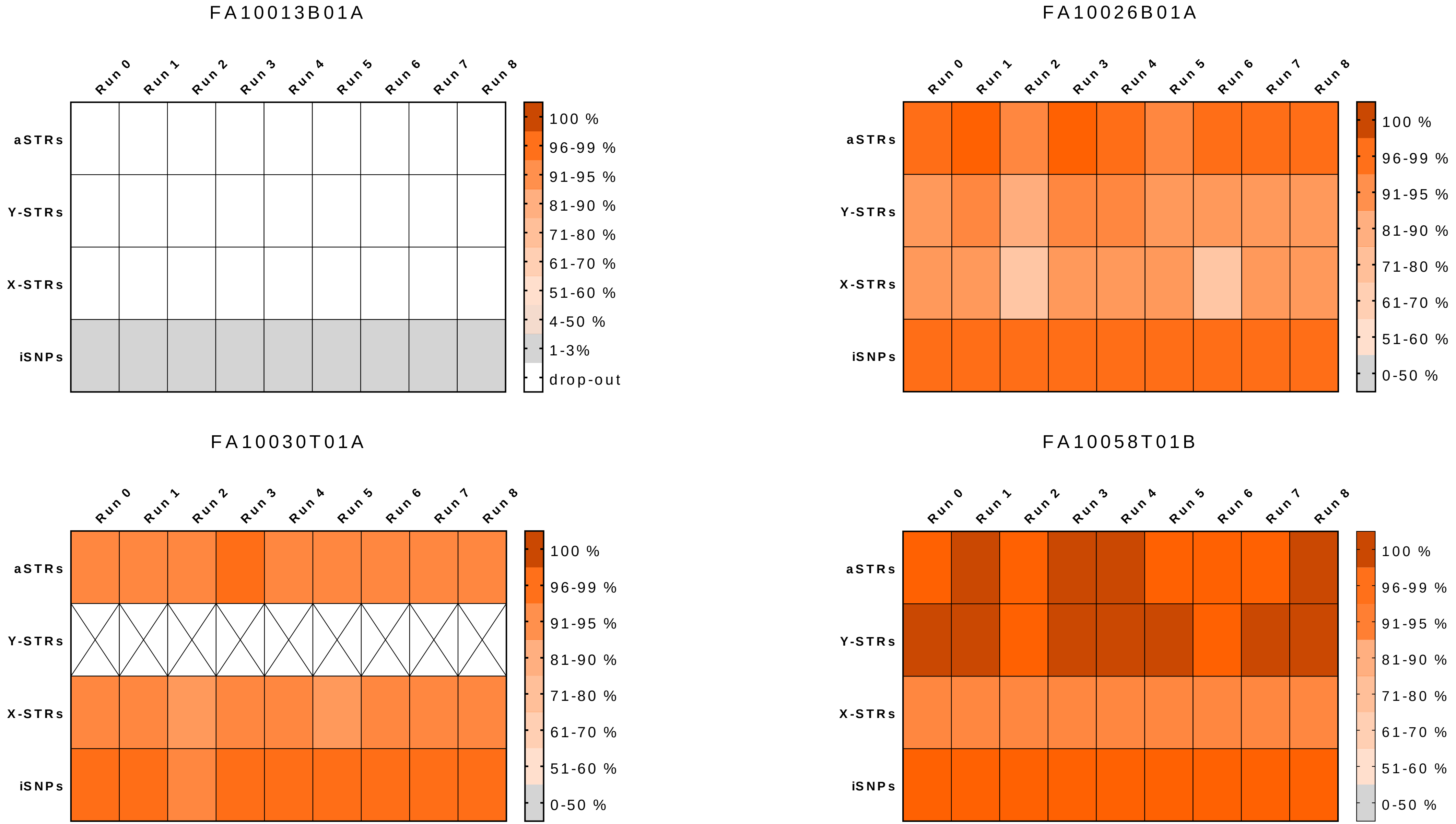

Figure S3. Ancient DNA heat maps for 27 autosomal STRs (aSTR), 24 Y-STRs, 7 X-STRs and 94 identity SNPs (iSNP) showing the percentage of concordantly typed forensic markers between all sequencing runs. Color gradient indicates the percentage of successfully typed alleles from 100% (intensive orange) down to ≤ 50% (light-grey), except for FA10013B01A. Color gradient for FA10013B01A indicates the percentage of successfully typed alleles 1-3% (light-grey) and drop-out (white).

Müller P., Sell C., Hadrys T., Hedman J., Bredemeyer S., Laurent F.X., Roewer L., Achtruth S., Sidstedt S., Sijen T., Trimborn M., Weiler N., Willuweit S., Bastisch I., Parson W., and the SeqForSTR-Consortium. Inter-laboratory study on standardized MPS libraries: Evaluation of performance, concordance and sensitivity using mixtures and degraded DNA
