# Supplementary material for: Inter-laboratory study on standardized MPS libraries: evaluation of performance, concordance, and sensitivity using mixtures and degraded DNA
Source: Int J Legal Med. 2019 Nov 19;134(1):185–98. doi: 10.1007/s00414-019-02201-2 (PMC6949318; doi:10.1007/s00414-019-02201-2)
Supplement: Supplementary file 4 — (PDF 78 kb) [file 414_2019_2201_MOESM4_ESM.pdf]

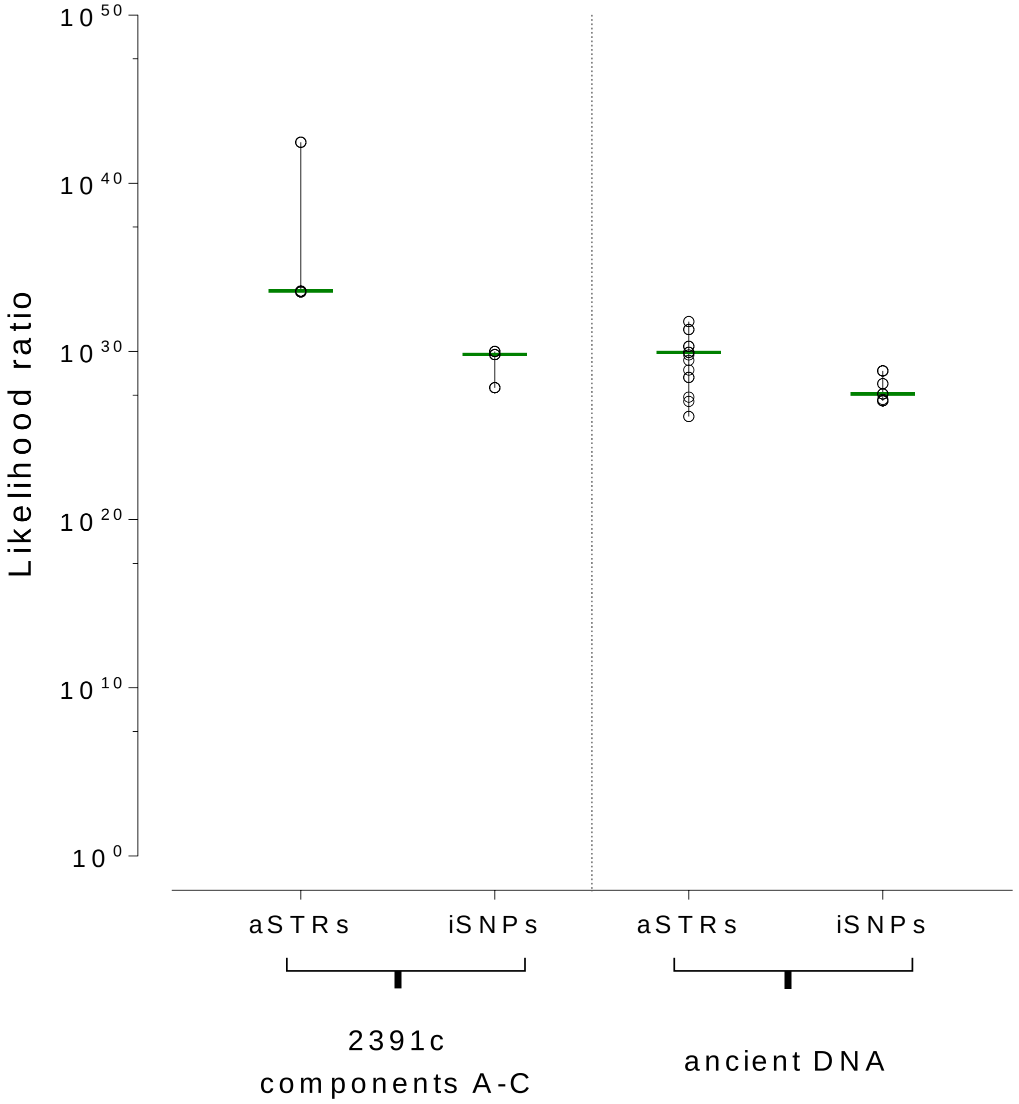

Figure S4. Likelihood ratios (LR; green line at median) showing the performance range of autosomal STRs (aSTR) and identity SNPs (iSNPs). LRs were calculated for NIST reference material 2391c (components A-C) and ancient DNA samples FA10026B01A, FA10030T01A and FA10058T01B. Allele frequencies for 27 aSTRs and 49 iSNPs were taken from SPSmart v5.1.1. (*Amigo et al.,* 2008; *Amigo et al.,* 2009). Only a partial fraction of the entire 94 iSNPs that were included in the validated SNPforID 52-plex panel (*Sanchez et al.,* 2006) was selected for LR calculation. Allele frequencies were selected according to the manufacturer´s provided information of geographic origin of the respective DNA sample (2391cA: European; 2391cB: American; 2391cC: Oceania). Allele frequencies for ancient DNA samples were taken from SPSmart v5.1.1 (*Amigo et al.,* 2008; *Amigo et al.,* 2009; European population).
