# Supplementary material for: Inter-laboratory study on standardized MPS libraries: evaluation of performance, concordance, and sensitivity using mixtures and degraded DNA
Source: Int J Legal Med. 2019 Nov 19;134(1):185–98. doi: 10.1007/s00414-019-02201-2 (PMC6949318; doi:10.1007/s00414-019-02201-2)
Supplement: Supplementary file 5 — (PDF 82 kb) [file 414_2019_2201_MOESM5_ESM.pdf]

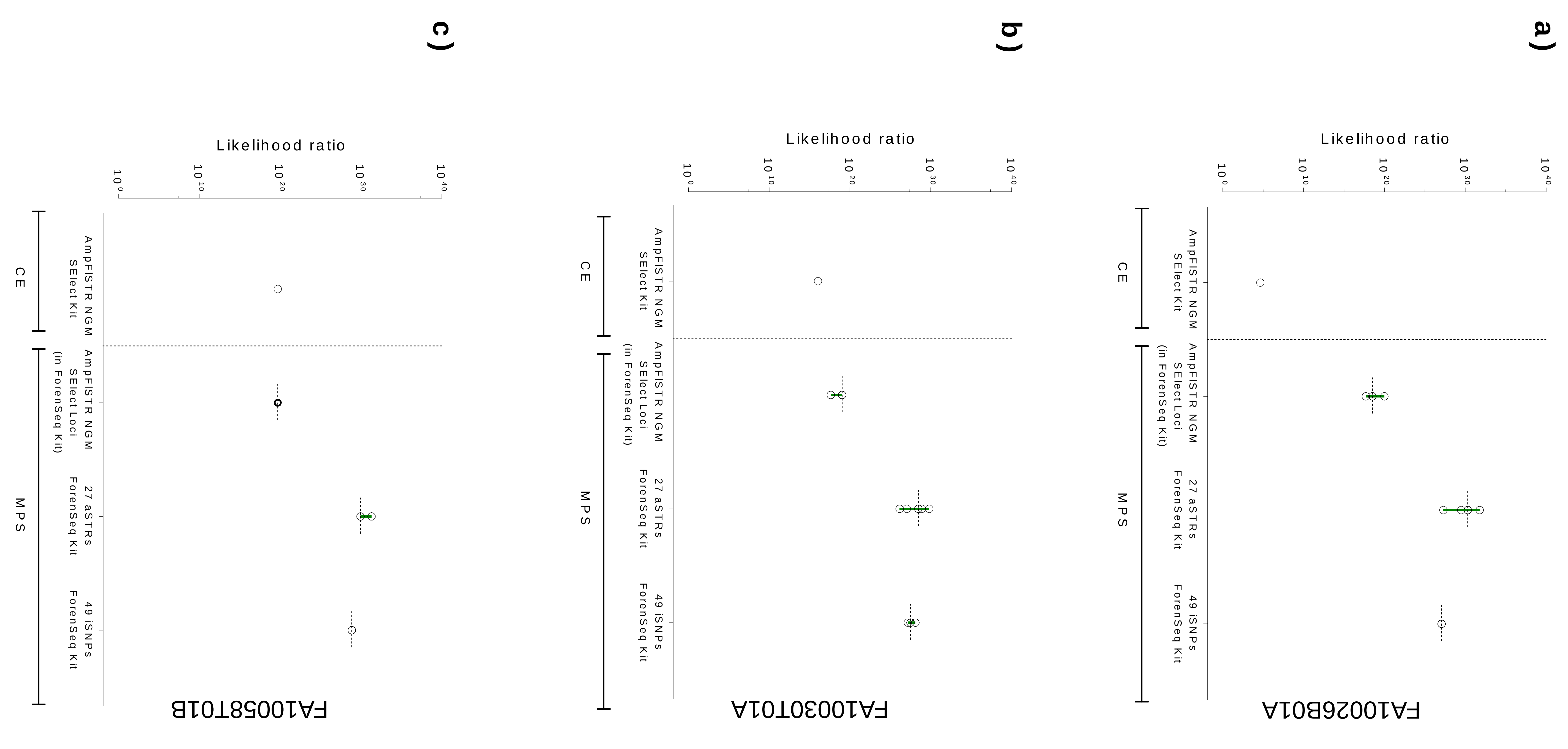

Figure S5. Comparison of CE-based and MPS-based genotyping for ancient DNA samples **a)** FA10026B01A, **b)** FA10030T01A and **c)** FA10058T01B. Likelihood ratios (LR) were calculated for the AmpFlSTR NGM SElect Kit, the AmpFlSTR NGM SElect loci as included in the ForenSeq DNA Signature Prep Kit and for the entire set of autosomal as well as for a subset of 49 identity SNPs (iSNPs) included in the latter kit. Orbitals portray LR estimates per sequencing run, green lines represent LR ranges and dashed lines show the median. Capillary electrophoresis analysis was able to successfully call 4, 12 and 15 STR genotypes for FA10026B01A, FA10030T01A and FA10058T01B, respectively. Samples were analysed with CE at the organising laboratory using the AmpFlSTR NGM Select kit (TFS). European population allele frequencies were taken from SPSmart v5.1.1. (*Amigo et al.,* 2008; *Amigo et al.,* 2009).

Müller P., Sell C., Hadrys T., Hedman J., Bredemeyer S., Laurent F.X., Roewer L., Achtruth S., Sidstedt S., Sijen T., Trimborn M., Weiler N., Willuweit S., Bastisch I., Parson W., and the SeqForSTR-Consortium. Inter-laboratory study on standardized MPS libraries: Evaluation of performance, concordance and sensitivity using mixtures and degraded DNA
